# Food Availability Cannot Explain Dispersal and Settlement Decisions of a Grassland Bird

**DOI:** 10.1002/ece3.74173

**Published:** 2026-08-09

**Authors:** Emily J. Williams, Theo K. Michaels, W. Alice Boyle

**Affiliations:** ^1^ Division of Biology Kansas State University Manhattan Kansas USA; ^2^ Department of Biology Western University London Ontario Canada

**Keywords:** breeding dispersal, fueling rates, nomadism, prey abundance, territory switching, within‐season movements

## Abstract

Food abundance is often postulated to shape animal habitat selection and movements. Those behaviors are critical to life‐history variation and making informed management decisions. However, the importance of food in shaping animal behavior is more often assumed than empirically tested. In mid‐continental grasslands, Grasshopper Sparrows ( *Ammodramus savannarum* ) display remarkable rates of breeding dispersal, with many individuals sequentially occupying distinct territories within a single breeding season. We tested the hypothesis that within‐season breeding dispersal and the iterative settlement decisions resulting from those movements are shaped by spatio‐temporal variation in food abundance. Working in an experimentally managed tallgrass prairie in Kansas in 2013–2015, we collected arthropod sweep samples as a measure of food availability (2014) and measured plasma metabolite concentrations to assess short‐term foraging dynamics and management of energy stores. We related those metrics to Grasshopper Sparrow territory densities and individual settlement patterns. As predicted, arthropod prey abundance varied seasonally. However, variation in prey did not correlate with metabolite concentrations, and neither prey nor metabolite concentrations were related to sparrow territory densities. Furthermore, metabolite concentrations of sparrows measured following dispersal were not elevated relative to those of sedentary individuals. Our results refute the hypothesis that food abundance explains the behavioral choices of a highly mobile grassland bird and implies that breeding dispersal is shaped by other ecological drivers. Our results underscore the need for critical tests of food‐related hypotheses regarding the causes of animal movements and habitat selection. The importance of doing so is especially urgent in rapidly declining species, for which effective management is critical.

## Graphical abstract

We test a commonly assumed explanation for animal movements—namely that food availability shapes where animals disperse to. Based on the results of four strong predictions, 3 years of data, and hundreds of individual birds, we show that food cannot explain breeding dispersal in this system, and we propose alternative explanations consistent with our data.

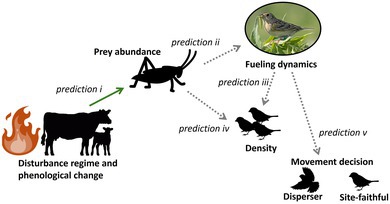

## 1 Introduction

Individual decisions about where to settle and breed define species' distributions and can reveal the ecological factors most strongly affecting vital rates (Pulliam and Danielson 1991). However, for most species, our understanding of habitat requirements rarely goes beyond associations with vegetation types. Even when we can describe landscape features associated with occupancy more precisely, it is uncommon that studies identify mechanistic links between habitat and demographic processes (Ceresa et al. 2020). Habitat selection is a multi‐scale process with outcomes influencing survival and reproduction (McGarigal et al. 2016). Lack of mechanistic knowledge of the factors underlying settlement decisions limits the ability to interpret interspecific interactions, predict responses to changing environmental conditions, and may lead to management that does not advance conservation goals.

Mobile animals are capable of making many, sequential settlement decisions over their lifetimes, reflecting changing requirements of different life stages and spatio‐temporal variation in habitat quality. In species undergoing annual return migrations, individuals select breeding habitat at least once each year (Zweifel‐Schielly et al. 2009). In many long‐distance avian migrants, site fidelity is very high (Cresswell 2014; Winger et al. 2019). In a few species, however, individuals undertake repeated breeding dispersal movements, making multiple settlement decisions within and among years (e.g., Botsch et al. 2012; Seifert et al. 2016; Becker et al. 2018). This iterative process permits animals to respond to changing environmental conditions (Jenkins et al. 2019; Macías‐Duarte and Conway 2021). Consequently, species in which dispersal is common provide excellent opportunities to determine which factors shape movement and settlement decisions, as animals' choices likely reflect anticipated fitness payoffs of current conditions (Shaw 2020).

Ecologists commonly invoke food limitation to explain many behaviors including movements, settlement choices, and resulting habitat associations (e.g., Steenhof et al. 2005; Slezak et al. 2024; Mueller et al. 2025). Access to sufficient food is clearly a crucial driver of habitat selection (Cody 1985). Nevertheless, at least three important considerations make the careful evaluation of food‐based hypotheses important. First, interactions between food availability and predation risk, sometimes modulated by the social environment, have long been recognized as shaping habitat suitability due to fitness trade‐offs between risk and energetic demands (Fretwell and Lucas 1969). Second, simple abundance of prey does not always translate into food availability; vegetation structure can influence access to food by shaping foraging substrate (Styring and bin Hussin 2004). Third, other factors may be more important than food in shaping animal behaviors; vegetation structure often determines the availability of den or nest sites (Norris et al. 2002), refugia from predators (Klug et al. 2010), and microclimates conducive to survival (Scheffers et al. 2014).

Mid‐continental grasslands are highly spatially and temporally variable due to pronounced interannual fluctuations in growing season rainfall that affect vegetation structure and productivity (Knapp and Smith 2001). In addition to temporally dynamic climatic conditions, the disturbances of grazing, fire, and their interactions result in marked spatial variation in primary productivity with consequences for higher trophic levels (Hertzog et al. 2016; Meyer et al. 2019). Phenological changes are also particularly strong in grasslands, where most aboveground biomass is re‐grown every year, leading to strong seasonal changes in most ecological processes. Unsurprisingly, arthropod and vertebrate communities both exhibit strong interannual fluctuations in abundance related to climatic variation (Joern and Laws 2013; Bruckerhoff et al. 2020). While spatial and temporal heterogeneity of vegetation structure is known to affect the composition of grassland bird communities and fitness (Johnson et al. 2019; Shew et al. 2019; Davis et al. 2020), we do not know whether food availability or some other factor shapes avian breeding dispersal behavior.

Birds living in mid‐continental grasslands are highly mobile. Most are migratory, and many are multi‐brooded species that exhibit between‐year and within‐year breeding dispersal (Jones et al. 2007; Dornak et al. 2013). Consequently, their settlement choices are likely to reflect conditions at breeding sites with anticipated fitness payoffs of reproductive success. However, breeding habitat selection is often measured only once within the breeding season (e.g., Henderson and Davis 2014; Skagen et al. 2018; Hardy et al. 2020) and, thus, may fail to capture responses to either spatio‐temporal variation in the external environment, or internal changes in bird requirements, or both (Forrest 2022).

**FIGURE 1:**
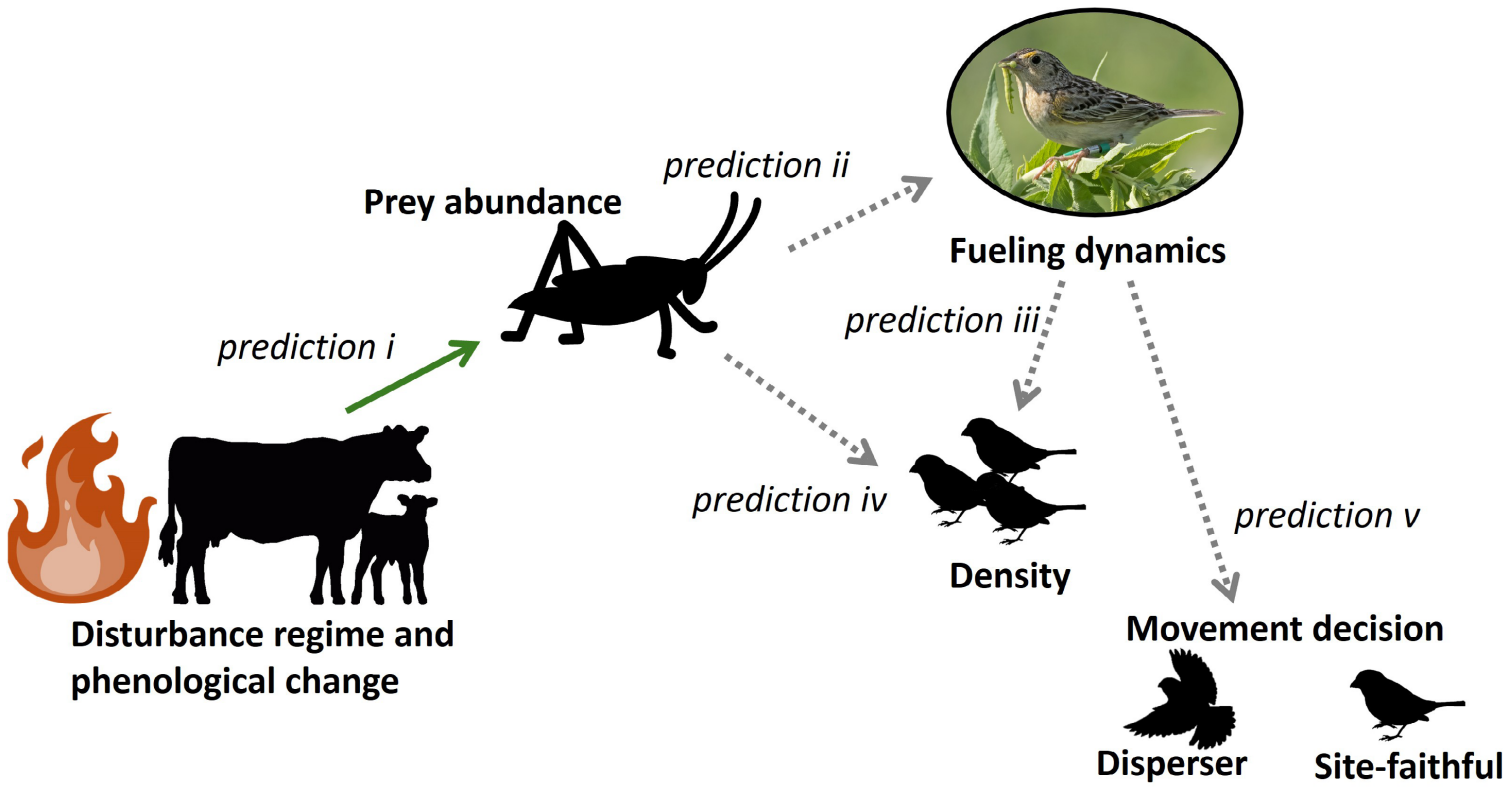
Summary of the hypothesized relationships tested in this manuscript. The solid green arrow (prediction *i*) represents the only relationship for which data were consistent with prediction. Dotted gray lines represents predictions unsupported by the data.

We tested predictions of food‐related explanations for the iterative settlement decisions in a grassland songbird exhibiting frequent within‐season breeding dispersal. Working in an experimentally managed tallgrass prairie in northeastern Kansas, we studied Grasshopper Sparrows ( *Ammodramus savannarum* ), monitoring territories and movements over three breeding seasons. To measure spatial and temporal variation in arthropod prey, we collected sweep samples three times over a breeding season. Additionally, we estimated fueling rates using plasma lipid metabolite assays (Guglielmo et al. 2005). A fundamental assumption of the food availability hypothesis is that food indeed changes across the breeding season. Thus, we expected that (*i*) food abundance would vary temporally over single breeding seasons and spatially in response to the individual and interactive disturbances of burning and grazing. We then predicted that if settlement decisions are shaped by food, (*ii*) fueling rates would be positively associated with time‐ and location‐specific abundances of prey, (*iii*) territory densities would be positively associated with fueling rates, and (*iv*) territory densities would be positively associated with the abundance of potential prey. Furthermore, if movement decisions are shaped by access to food, individual‐level (*v*) fueling rates would increase from first to second territories to a greater extent in birds that dispersed relative to site‐faithful individuals (Figure 1).

## 2 Materials and Methods

### 2.1 Study Species and Site

Grasshopper Sparrows are ~17 g grassland‐dependent songbirds distributed from southern Canada to much of the United States, Mexico, Caribbean, and parts of Central and South America (Vickery 2020). In tallgrass prairies of the Central Great Plains of the United States, Grasshopper Sparrows commonly breed in areas managed with a 2‐ to 3‐year burn frequency in combination with low‐intensity grazing (Powell 2008). Periodic burning helps control the growth of woody vegetation while allowing dead grass to accumulate that Grasshopper Sparrows use to construct their domed ground nests. Grazing produces patches of low vegetation suitable for foraging. Grasshopper Sparrows typically arrive in Kansas during April and establish territories soon after. Females arrive ~5 days after males with pairs forming in late April and clutch initiation beginning in early May. Grasshopper Sparrows can produce at least two broods per year, and each successful breeding attempt takes ~21 days (first egg to fledge) in Kansas. Young typically disperse away from natal territories beginning soon after fledging (Anthony et al. 2013). Most birds depart on southward migrations by early October (Hill and Renfrew 2019). Adult Grasshopper Sparrows consume mainly arthropods during the breeding season (~70% invertebrates, 30% seeds), preferring grasshoppers (Orthoptera), Arachnids, beetles (Coleoptera), and caterpillars (Lepidoptera); over a third of the arthropod portion of diets consists of grasshoppers in the family Acrididae (Kaspari and Joern 1993). Grasshopper Sparrows only feed arthropods to nestlings (Kaspari and Joern 1993).

We studied Grasshopper Sparrows between April and August from 2013–2015 in the Flint Hills of northeastern Kansas. We worked at the Konza Prairie Biological Station (hereafter, “Konza”), a 3487‐ha tract of tallgrass prairie co‐owned by Kansas State University and The Nature Conservancy, and at the adjoining Rannell's Flint Hills Prairie Preserve, a 1175‐ha tract owned by Kansas State University. We studied Grasshopper Sparrows in 18 pastures, managed with replicated experimental treatments of either year‐long bison grazing, cattle grazing, or no grazing, in combination with prescribed fires every 1–3 years: six cattle‐grazed pastures (April–October with cow/calf pairs), patch‐burned on a staggered 3‐year rotation, two cattle‐grazed, burned annually (steers stocked at double the typical density from April to July; Owensby et al. 2008), two bison‐grazed, burned annually, two bison‐grazed, burned every 2 years, three ungrazed, burned annually, and three ungrazed, burned every 2 years. Due to the multi‐year duration of these management approaches, vegetation within upland regions of pastures managed in the same ways are similar to one another; in all pastures, trees and shrubs are encroaching in drainages (Silber et al. 2024). Controlled burns are conducted at the site in the spring between March 1 and May 5 with the majority of burns completed before the beginning of the growing season and prior to the arrival of most migratory birds (i.e., by early‐mid April). Climate at the site is characteristic of eastern tallgrass prairie, with 835 mm mean annual precipitation and high interannual variability in rainfall (CV = 25%) and plant productivity (Knapp et al. 1998; Knapp and Smith 2001). Over 75% of annual precipitation falls during April–September and determines relative biomass of dominant grasses and forbs (Nippert et al. 2006; Raynor et al. 2015). Within each pasture, we randomly located a 10‐ha plot > 20 m from any road or fence line. Despite the fact that we located territorial sparrows within each of the pastures, the patch‐burned grazed pastures consistently provided the most extensive areas of vegetation structure that sparrows select (Silber et al. 2024).

### 2.2 Bird Capture, Densities, and Movement

We captured birds throughout the breeding season by placing a mist‐net (12 m long, 38 mm mesh, Ecotone, Gdynia, Poland) adjacent to favored perches within territories and by luring birds using conspecific territorial song. We placed a numbered USGS band and unique combinations of three colored leg bands to enable subsequent individual visual recognition. We sexed birds by the presence of cloacal protuberances (males) or brood patches (females) and took standard morphometric measurements. Territorial male behavior included singing, wing flicking displays, and initiation of aggressive chases with other males (Vickery 2020). Female Grasshopper Sparrows do not sing, spend most of their time on the ground (Vickery 2020), and we usually detected them only after locating nests. For these reasons, the sample size of females was low; we included them in analysis of the relationship between food availability and fueling rates, but did not explicitly test for sex‐specific behavior in this study. In analyses involving territory density, we restricted the dataset to males as they are the territorial sex. We determined locations of territorial males by repeatedly re‐sighting color‐banded birds throughout the breeding season. We conducted surveys during morning hours beginning as early as 30 min prior to sunrise and lasting an average of 90 min (20–400 min range with duration varying depending on the number of birds and size of pasture). We conducted surveys roughly weekly (interval between surveys: mean = 8.4 days, SD = 3.1, range = 6–17 days). We ensured that we carefully covered all areas within the 10‐ha plot each survey and based density estimates on those data. We located as many of the singing males outside of the plot as possible to determine dispersal distances and identify unbanded males that we then captured. Because some pastures were large (up to 226 ha), we were unable to thoroughly cover every pasture in every survey.

To estimate spatial and temporal variation in densities of Grasshopper Sparrows (predictions *iii* and *iv*), we mapped territories of all marked and unmarked individuals, recording the location of each individual with GPS units (Garmin, Olathe, KS, USA). We also searched for banded individuals elsewhere within each pasture, collecting two to eight GPS locations per individual per survey. We divided breeding seasons into early, mid, and late time periods, conducting three to five surveys per period. Dates of each period varied slightly among years due to differences in bird phenology and the timing of field efforts; the “early season” period began from April 25 to May 12 and ended from May 28 to June 18, the “mid season” period began from May 30 to June 19 and ended from June 20 to July 2, and the “late season” period began from June 22 to July 2 and ended from July 31 to August 12. Birds initiated new nests throughout all three periods of the breeding season and our field efforts concluded prior to the post‐breeding molt period. We estimated year‐ and period‐specific densities as the mean number of unique territorial males within each 10‐ha plot. Where territories spanned plot boundaries, we counted these as half a territory. On 19 individuals, we affixed 0.52–0.56 g radio‐transmitters with detection ranges from 0.8 to 1.6‐km. Transmitters and harnesses weighed ≤ 4% body mass. We confirmed the location of tagged males every 2 days. If they disappeared from initial territories, we searched until we relocated them, or for 2 weeks over a ≥ 5‐km radius. We failed to locate three tagged birds, inferring them to have died or dispersed beyond our search radius.

We considered individuals to have dispersed if they (1) displayed territorial behavior ≥ 100 m away from the centroid of their previous known territory or nest location or (2) were not re‐sighted at their initial territory ≥ 1 week after their nest failed or fledged. We chose the 100 m cutoff because Grasshopper Sparrow territory sizes are on average (±SE) 43 ± 2 m in diameter at our site (Williams and Boyle 2018). Thus, a shift of 100 m represents a movement of > 2 territory widths. Conversely, we considered individuals to be site‐faithful if they (1) continued to display territorial behavior within 100 m of their original territory or nest or (2) were re‐sighted again on their initial territory > 1 week after their nest failed or fledged. We used a combination of color band re‐sighting and radio‐telemetry to determine settlement locations following dispersal movements (Williams and Boyle 2018).

### 2.3 Arthropod Sampling and Processing

To determine the spatial and temporal patterns of food availability, we collected sweep samples in the last 2 weeks of each month in May, June, and July of 2014 corresponding to the early, mid, and late periods. We sampled arthropod prey along three 80‐m transects within each pasture, centered at each of three randomly located points within grassland habitat. One point in each pasture was located within the 10‐ha plot, and two were located outside the plot, and we constrained points to be ≥ 100 m from any other point. We swept the tops of herbaceous plants and sparsely vegetated areas where Grasshopper Sparrows typically forage using a heavy duty sweep net (Bioquip; 46 cm diameter, 91.5 cm handle). Each sweep sample consisted of the contents of 200 “sweeps” of the net along each transect covering 150 m^2^ per transect (80 m × 1.88 m sweep width). We immediately placed net contents in plastic bags, stored them on ice in the field, and froze at −20°C within ~4 h of collection. We followed Konza LTER protocols (Joern 2025) by sampling between 9:00 and 15:00, on calm days (wind ≤ 15 mph) when cloud cover was ≤ 50%, and between 25°C and 40°C temperatures.

We carefully separated arthropods from debris, measured potential prey items to the nearest 5 mm, counted all individuals, and identified arthropods to Order. We estimated arthropod abundance and calculated the “edible mass” of potential prey based on the Orders known to be important food for Grasshopper Sparrows (Joern 1988) by weighing all Orthoptera, Hemiptera, Lepidoptera, Coleoptera, and Arachnids together. In preliminary analyses, we explored using abundance (*n* arthropods) rather than mass; because results were similar, we proceeded with a single metric (edible biomass) and did not explore other metrics such as dry mass in subsequent analyses. Due to the immense time and effort required to collect and process these data, our inferences regarding predictions *ii* and *iv* are limited to 1 year.

### 2.4 Blood Sampling and Processing

Triglycerides (TRIG) are blood plasma lipid metabolites that provide measures of energy dynamics, with high concentrations reflecting high food intake rates, fat deposition, and mass gain (Jenni‐Eiermann and Jenni 1994). Upon first capturing adult birds, we collected a blood sample within 5 min of capture (Guglielmo et al. 2002). We also attempted to recapture males known to have dispersed to compare TRIG concentrations between subsequent territories. We collected ~70–100 μL of blood from the brachial vein in heparinized capillary tubes and stored them on ice, centrifuging them within ~6 h. We stored plasma at −80°C and analyzed metabolite concentrations using a colorimetric endpoint assay (Sigma reagents F6426 (A), T2449 (B), and G7793) run on an Eon microplate spectrophotometer (Biotek Instruments Inc.). We diluted plasma 1:1 with saline and ran assays in duplicate using 5 μL diluted plasma and 240 μL and 60 μL of reagents A and B, respectively, warmed to 37°C, and measured absorbance at 540 and 750 nm (Guglielmo et al. 2002).

### 2.5 Analyses

We sought to make inferences at the population level, and the scale at which our factors were applied varied depending on prediction (Table 1). We performed all analyses in R 4.3.1 (R Core Team 2023). To test predictions of the food availability hypothesis to explain movement and settlement decisions of Grasshopper Sparrows, we either employed linear mixed effects models (LMMs, prediction *i*), linear models (LMs, predictions *ii–iv*), or a one‐tailed *t*‐test (prediction *v*), as described below. For linear models, we used the *lme4* package (Bates et al. 2014). We *ln*‐transformed estimates of edible biomass of potential prey and TRIG concentrations and square‐root transformed territory densities to improve homogeneity of residuals. To test each prediction, we evaluated main effects, and when relevant, their interactions, confirming that each model met assumptions using Shapiro–Wilk tests and visualization in *ggResidpanel* (Goode and Rey 2025). When relevant, we compared potential models using AICc (Burnham and Anderson 2002) with the *AICcmodavg* package (Mazerolle 2023). We interpreted overall model fit using AIC and/or *R* ^2^ values (marginal and conditional *R* ^2^ in LMMs) using the *performance* package (Lüdecke et al. 2021). We interpreted relationships among variables using the sign and strength of beta coefficients from the best‐fit model.

**TABLE 1:** Replicates and level of inference for each prediction.

Prediction	Scale of inference	Scale at which factor applied	Number of replicates
Prediction *i*	Monthly at pasture scale	Pastures	6 sweep locations/pasture in 3 time periods (within one summer) in 18 pastures = 324
Prediction *ii*	Monthly at pasture scale	Pastures	238 individuals (within 18 pastures × 3 time periods = 54)
Prediction *iii*	Monthly × yearly at pasture scale	Pastures	661 individuals (within up to 18 pastures × 3 time periods × 3 years = 162)
Prediction *iv*	Monthly at pasture scale	Pastures	Territory densities in 18 pastures × 3 time periods = 54
Prediction *v*	Individuals	Individuals	*N* = 23

For food availability to drive dispersal, it must vary in space and time. Thus, we first determined the spatio‐temporal patterns in food abundance within 2014 (prediction *i*), relating variation in mean biomass of edible arthropods within pastures (hereafter mean edible biomass) to time period (early, mid, and late season), grazing treatment (cattle, bison, ungrazed), and years since burn (0, 1, 2). We evaluated a model that included all three additive effects, as well as models including the period × grazing and period × years‐since‐burn interactions. In all LMMs, we included random effects to account for repeated sampling of the transects within pastures over the season.

We then assessed if variation in prey abundance translated into differences in fueling dynamics of sparrows. We used LMs to assess if TRIG was positively associated with the spatial and temporal variation in mean edible biomass (prediction *ii*). These models included both male and female adult sparrows from 2014 to match arthropod collection efforts. We removed two outliers from the data whose TRIG values (14.626 and 6.776 mmol/L) were well beyond the standard curves in lab assays and therefore unreliable. Additionally, because TRIG typically varies over the course of the day and with endogenous factors (Hays 2008), we first determined if capture time, capture date, fat score, and sex were associated with *ln*‐TRIG in our study. All four variables were strongly correlated with TRIG; thus, we included them as covariates in the main model of the relationship between TRIG and mean edible biomass.

Next, if food and fueling capacity shapes post‐dispersal settlement, we expected territory densities to be positively associated with fueling rates (prediction *iii*). We extracted data for territorial males for which we obtained TRIG data (spanning all years of the study). Because *ln*‐TRIG varied with temporal and physiological factors in prediction *ii*, we first constructed LM models with *ln*‐TRIG as the response variable predicted by capture time, capture year, and fat score. As this analysis was restricted to territory males, we did not account for sex in models. We also did not include capture date because territory densities were estimated at the temporal scale of seasonal time periods. Thus, we reevaluated the best‐fit covariate model and extracted residual *ln*‐TRIG, relating those residuals to pasture‐ and period‐specific densities of sparrow territories.

Even where food availability varies spatially, fueling may not reflect that variation if birds are able to compensate behaviorally by adjusting foraging effort to meet their energetic demands. Thus, we also determined if territory densities were positively associated with the abundance of mean edible biomass which we estimated at the pasture level (prediction *iv*). In this analysis, we again restricted data to the year (2014) in which we collected arthropod samples. We evaluated models of mean sparrow territory density predicted by mean edible biomass, including time period, and the interaction of mean edible biomass and time period. For predictions *ii–iv*, we accounted for the temporal and spatial variability in food availability, territory density, and TRIG levels by matching the location timing of each metric to the other in analyses. For example, in prediction *iii*, we related the TRIG values of any male captured in a specific pasture to the corresponding territory density of that same pasture during the same time period of the same year.

Finally, we predicted that if the reason for sparrow dispersal was to seek areas with improved foraging conditions, we would see the fueling rates of individual dispersed birds increase from first to second territories, whereas those that remained in a single location would not increase or not increase to the same extent over the same period (prediction *v*). We first identified adult males that had dispersed (*n* = 12) and those that were site‐faithful (*n* = 11) for which we had measures of TRIG associated with first and second territories. We calculated residual *ln*‐TRIG values (assessing models that included capture time, year, and fat score) and subtracted the value associated with the first territory from the second. Positive differences indicated an increase in fueling rates from first to second territories, whereas negative differences indicated the reverse. We tested if this difference in residual *ln*‐TRIG was higher in dispersers than site‐faithful individuals in a one‐tailed *t*‐test. We summarize our analyses for each prediction in Table S1.

## 3 Results

Over three breeding seasons, we captured and color‐banded 779 adults (647 males, 132 females). From re‐sighting and radio‐telemetry efforts, we recorded dispersal for 220 of 779 birds (28.2%; 213 males, 7 females). We analyzed 614 blood samples for plasma metabolites over all 3 years and collected 324 sweep samples in 2014 (Table 1).

**FIGURE 2:**
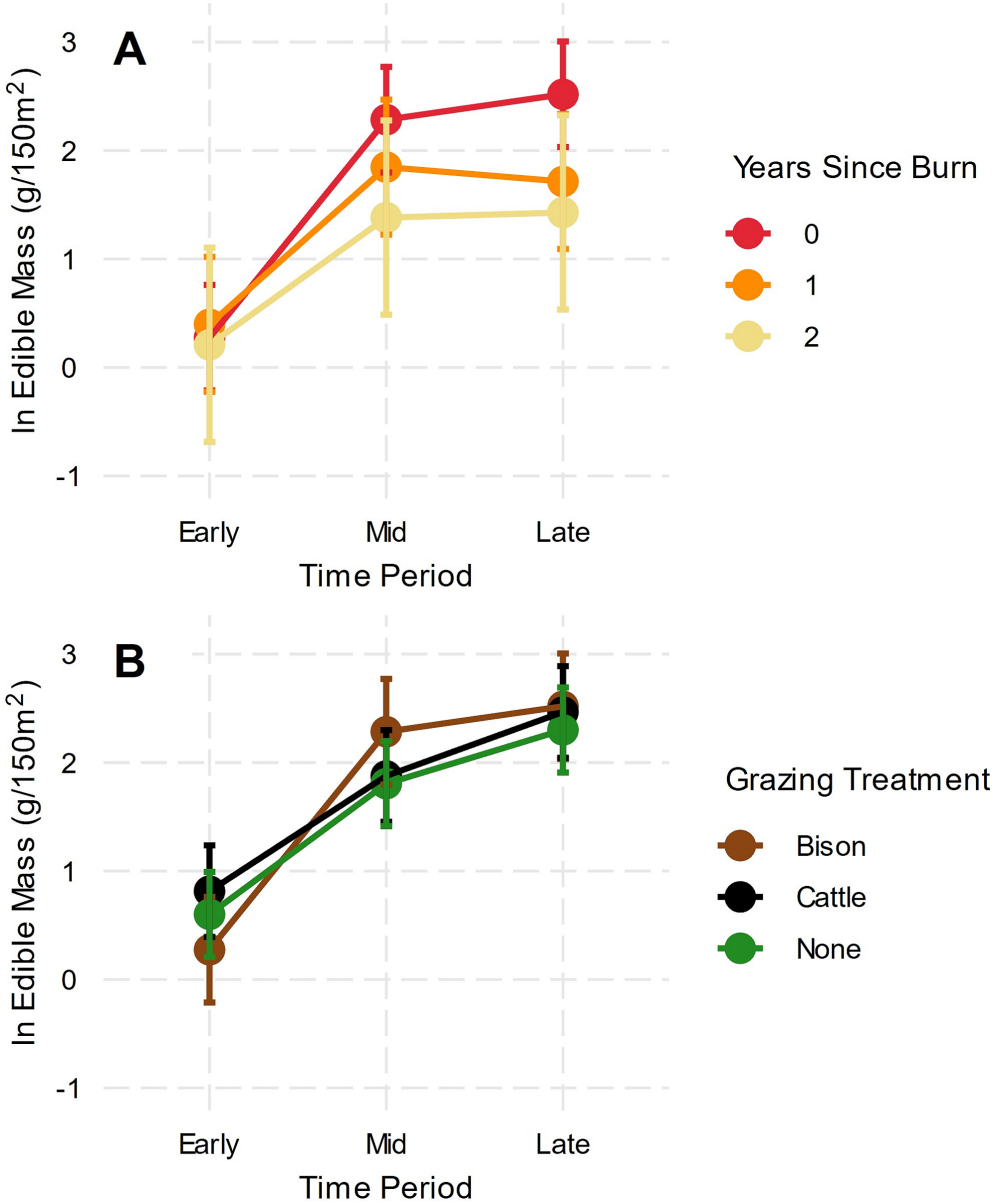
The edible biomass of arthropod prey available to foraging Grasshopper Sparrows increases seasonally, and does so in ways that depend upon fire (panel A) and grazing regimes (panel B).

As expected, food availability for sparrows varied substantially, both spatially and temporally over the 2014 breeding season (prediction *i*; Figure 2). The edible biomass of arthropods in samples increased more than fourfold over the season, varying from a mean of 2.52 (±0.28 SE) to 10.3 (±0.93 SE) g/150 m^2^ from early to late periods. The model containing interactions between time period and grazing, and between time period and year of burn was a far better fit to the data than the model of only additive main effects (ΔAIC_c_ = 19.05; Table S2). This model had a marginal *R* ^2^ of 0.57 and a conditional *R* ^2^ of 0.74. Food availability increased considerably from early‐ to mid‐season, and then in some pastures, increased to a smaller extent between mid‐ and late‐season periods (Figure 2). Time since fire influenced the degree to which edible biomass increased across the season. While edible biomass was relatively similar early in the season regardless of when pastures were last burned, pastures that burned in the current year had the highest edible biomass, followed by pastures that burned one season prior, and pastures that burned 2 years prior had the lowest biomass (Figure 2A). Seasonal change in edible biomass also varied somewhat among grazing treatments, with cattle‐grazed pastures having the highest edible biomass early in the season and bison‐grazed pastures having the highest edible biomass mid‐season to late season (Figure 2B).

**FIGURE 3:**
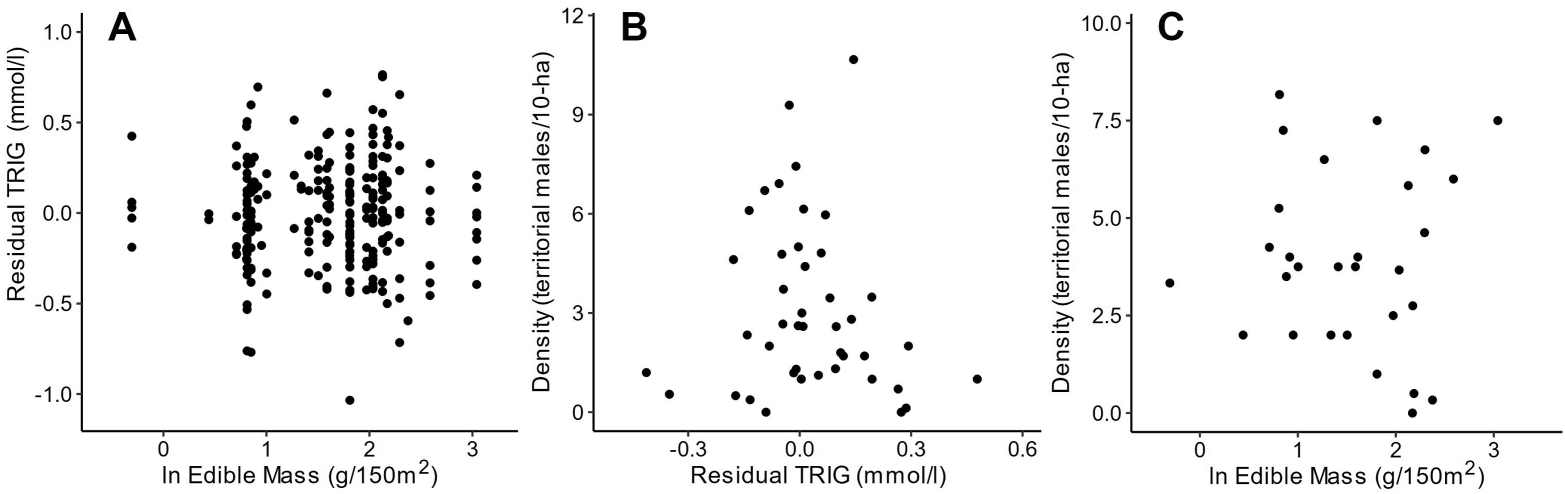
Results showing (A) no relationship between the edible biomass of prey and residual TRIG (accounting for capture time, capture date, fat score, and sex; prediction *ii*), (B) residual TRIG (accounting for capture time and capture year) and male sparrow territory densities (prediction *iii*), and (C) edible biomass of prey and male sparrow territory densities (prediction *iv*).

To determine if sparrow foraging responded to the spatio‐temporal variability in arthropod prey abundance, we related the 2014 edible biomass to concentrations of TRIG, matching samples from sparrows to the pasture and time period within 2014 in which they were captured (prediction *ii*). The model containing all putative covariates (capture time, capture date, fat score, and sex) explained 37% of the variation in *ln*‐TRIG (whole model *F*_9,231_ = 15.1, *p* < 0.0001). As expected, TRIG increased over the day, over the season, was lower in males than females and was highest in the fattest individuals (Table 2). After accounting for these factors, *ln*‐TRIG was unrelated to *ln*‐edible biomass (partial effect test, *p* = 0.909; whole model *R* ^2^ = 0.37, *F*_10,227_ = 13.4, *p* < 0.0001; Figure 3A).

**TABLE 2:** Prediction *ii* model results: Results of linear model explaining Grasshopper Sparrow fueling rates (i.e., concentration of TRIG).

Model fit: *R* ^2^ = 0.37; adjusted *R* ^2^ = 0.34			
Parameter	Estimate (SE)	*t* statistic	*p* (>|*t*)
Edible biomass	−4.3e‐3 (0.04)	−0.11	0.909
Capture time	2.0e‐5 (2.8e‐6)	6.93	< 0.001
Capture date	3.4e‐3 (9.7e‐4)	3.57	< 0.001
Fat Score 0.5	0.04 (0.08)	0.54	0.590
Fat Score 1	0.04 (0.05)	0.91	0.362
Fat Score 1.5	−0.29 (0.15)	−1.90	0.059
Fat Score 2	−0.04 (0.07)	−0.58	0.564
Fat Score 3	0.14 (0.12)	1.16	0.249
Fat Score 4	0.36 (0.14)	2.59	0.010
Sex M	−0.21 (0.05)	−4.14	< 0.001

Using the 661 males over 3 years for which we obtained TRIG estimates, capture time and year were the combination of covariates that best explained variation in *ln*‐TRIG (Table 3). Fat score was not related to TRIG in these analyses (differing from prediction *ii*), likely because males generally carried little fat. Using residual *ln*‐TRIG extracted from this model matched to the time period and pasture in which males were captured, we determined whether fueling dynamics explained sparrow territory densities, and whether that relationship varied additively or interactively with time period (prediction *iii*). The best model did not include time period (Table 3; ΔAIC 4.41) nor the interaction term (ΔAIC 6.91), and the top model (residual TRIG alone) explained only negligible variation in sparrow territory densities (whole model *R* ^2^ = 0.01, *F*_1,41_ = 0.5, *p* = 0.489; Figure 3B).

**TABLE 3:** Prediction *iii* model results: Results of linear model explaining the mean density of Grasshopper Sparrow territories in relation to TRIG.

	Model 1: No covariates	Model 2: With time	Model 3: With interaction
AICc^a^	208.31	212.71	215.22
∆AICc^b^	0	4.41	6.91
AICcWt^c^	0.88	0.10	0.03

Model 1 fit: log likelihood = −100.8; deviance = 274.2; residual df = 41, *R* ^2^ = 0.01			
Parameter	Estimate (SE)		
ResidTRIG	−1.67 (2.4)	−1.59 (2.50)	−5.66 (4.13)
Time period mid		−0.47 (0.97)	−0.49 (0.97)
Time period late		−0.71 (0.99)	−0.66 (1.05)
ResidTRIG × mid			8.80 (5.61)
ResidTRIG × late			1.54 (6.76)

^a^ Akaike information criterion corrected for small sample size.

^b^ Difference in AIC_C_ between top model (1) and other models. Differences > 4 generally interpreted as strong evidence for distinguishing fit of models to data.

^c^ The relative likelihood that the model is the best among a set of candidate models, with a weight of 1.0 being the most likely.

In case TRIG reflected behavioral compensation to meaningful variation in arthropod prey, we determined whether arthropod prey from 2014 sweeps related to sparrow territory densities in that year, assessing models including time period and its interaction with edible biomass (prediction *iv*). Again, the best model did not include time period (Table 4; ΔAIC 4.41) nor the interaction term (ΔAIC 9.50), and the top model (edible biomass alone) did not explain sparrow territory densities (whole model *R* ^2^ < 0.01, *F*_1,27_ < 0.1, *p* = 0.985; Figure 3C).

**TABLE 4:** Prediction *iv* model results: Results of linear model explaining the mean density of Grasshopper Sparrow territories in relation to prey biomass.

	Model 1: No covariates	Model 2: With time	Model 3: With interaction
AICc^a^	136.34	140.74	140.47
∆AICc^b^	0	4.41	9.50
AICcWt^c^	0.89	0.10	0.01

Model 1 fit: log likelihood = −64.7; deviance = 147.0; residual df = 27, *R* ^2^ = < 0.01			
Parameter	Estimate (SE)		
Edible biomass	0.01 (0.59)	0.45 (0.73)	−0.05 (1.44)
Time period mid		−1.18 (1.25)	−0.53 (2.57)
Time period late		−1.33 (1.37)	−4.17 (3.05)
Edible biomass × mid			−0.15 (1.85)
Edible biomass × late			1.74 (1.94)

^a^ Akaike information criterion corrected for small sample size.

^b^ Difference in AIC_C_ between top model (1) and other models. Differences > 4 generally interpreted as strong evidence for distinguishing fit of models to data.

^c^ The relative likelihood that the model is the best among a set of candidate models, with a weight of 1.0 being the most likely.

**FIGURE 4:**
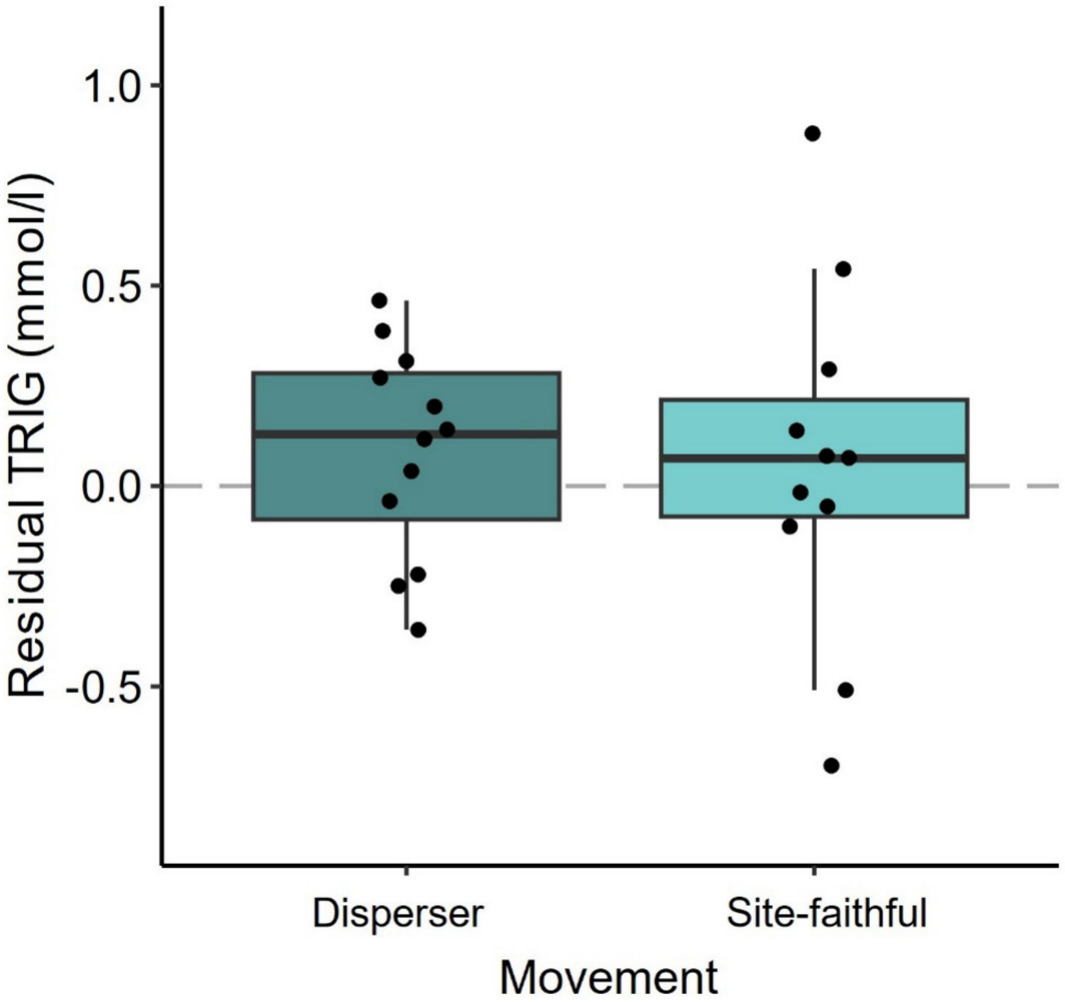
The change in residual TRIG (accounting for time of day and year; prediction *v*) did not differ among birds that dispersed to new territories (dark teal) compared to those that remained on the same territory (pale teal) during successive nesting attempts.

Finally, in paired comparisons of individual birds for whom we were able to collect TRIG samples during first and second territories (prediction *v*), TRIG did not increase, or increase more in birds that dispersed between successive territories compared to site‐faithful birds that remained on initial territories (one‐tailed *t*‐test of differences in residual TRIG; *t* = 0.2, df = 21, *p* = 0.416; Figure 4).

## 4 Discussion

We provide strong evidence that spatio‐temporal variation in food availability cannot explain the remarkable prevalence of within‐season breeding dispersal nor post‐dispersal habitat selection decisions made by Grasshopper Sparrows in tallgrass prairies of the Central Great Plains of North America. While the availability of potential prey sampled in 1 year did vary tremendously, seasonally and as a consequence of experimental disturbances (Figure 2), that variation in food availability did not translate into metrics reflecting sparrow foraging. This lack of association implies that, irrespective of variation in food availability, birds are able to meet their foraging needs wherever they locate their territories. Furthermore, we found no evidence that birds increased their prey consumption following dispersal relative to sparrows that remained on initial territories. It is very unlikely that our metrics of prey availability and fueling dynamics failed to capture biologically important relationships, as each of these metrics was clearly related to interpretable exogenous or endogenous factors. For example, arthropod prey increased strongly over the season and differed among pastures with distinct grazing and burning histories in ways that were consistent with previous work (Belovsky et al. 2004; Jonas and Joern 2007). Additionally, plasma metabolite concentrations reflected expected temporal patterns and relationships with body composition. Thus, we have confidence in the power of these methods to refute food availability as an explanation for dispersal and habitat selection in this system.

Why, then, do Grasshopper Sparrows disperse between breeding attempts? One potential explanation is that parents do indeed face food limitation during the breeding season, but they maintain constant intake rates themselves and pass on that variation to young in terms of variable food delivery rates. Thus, we could fail to see the association between variation in food availability and TRIG (prediction *ii*) that we expected. We strongly suspect that this explanation does not hold in this study system for several reasons. First, grassland obligate species at this site have among the fastest developmental rates described across the ranges of Grasshopper Sparrows, Eastern Meadowlarks ( *Sturnella magna* ), and Dickcissels ( *Spiza americana* ) (Winnicki 2019). Rapid growth rates are fueled by high food delivery rates which are inconsistent with this hypothesis. Second, elsewhere in Kansas, nestling metabolite concentrations, mass, and size metrics were unrelated to spatial variation in food availability as would be predicted by this hypothesis (Kraus et al. 2025). Third, while dispersers did experience lower nest success (Williams and Boyle 2019), over 10 years of nest data at this site, the majority of nest failures was due to predation (259 of 402 failed nests; 65%) while only 12% (50/402) were attributed to abandonment (unpublished data). If parents routinely passed on the costs of limited food to nestlings, then we would expect far more of the nest failures to be due to abandonment.

We conclude that other axes of variation in habitat quality (i.e., not food) must shape this behavior. We suspect that the ultimate driver is likely safe nest sites located in close proximity to suitable foraging substrate. Grassland‐dependent bird species generally, and Grasshopper Sparrows specifically, make territory selection decisions reflecting very nuanced preferences for specific vegetation structures (Dieni and Jones 2003; Sandercock et al. 2015; Silber et al. 2024). Associations between territory selection and disturbance or climate differ across species' ranges (Wilson et al. 2018), likely implying that birds' fitness depends on specific vegetation features that can be produced by various combinations of disturbance and climatic regimes. Furthermore, associations between both vegetation structure and rainfall, and between demography and rainfall, are lagged in this species, implicating indirect vegetation‐mediated drivers of sparrow population dynamics (Silber et al. 2023). If those indirect relationships are not mediated by food, then the availability of nest sites for Grasshopper Sparrows, which are constructed of dead vegetation left over from previous years, very likely influence dispersal and iterative habitat selection within a breeding season (Williams and Boyle 2019; Silber et al. 2023, 2024). Like other grassland birds in the Great Plains (Ludlow et al. 2014), Grasshopper Sparrows have very low nest success driven by high predation rates (Smith et al. 2024). Furthermore, they construct nests in ways that reflect adaptive responses to climatic challenges of ground nesting in highly exposed habitats (Corimanya et al. 2024). As grassland vegetation structure transforms itself over a growing season due to phenology, fire, and grazing, conditions that are well suited for successful reproduction must also be a moving target. Consistent with this hypothesis, dispersers in this system had lower nest success early in the season compared to site‐faithful birds, and dispersal functions in the context of increasing nest success following those movements (Williams and Boyle 2019).

Despite these alternate explanations for dispersal, it is still surprising that food availability is apparently so unimportant. For example, we know that Grasshopper Sparrows can depress local prey densities by up to 25% during the post‐fledging period (Joern 1986; Kaspari and Joern 1993) when juvenile birds are growing quickly. With clutches typically ranging from 3 to 5 (mean = 4.1, max 7) and incredibly fast developmental periods at this site (i.e., chicks fledge within 7 days of hatching), the parental foraging demands must be high. Indeed, food availability is central to theoretical models of alternative movement strategies (Andersson 1980). Likewise, food has repeatedly been demonstrated as a driver of dispersal in empirical studies in birds (e.g., Grande et al. 2009; Fernández‐Chacón et al. 2013; Nicolaus et al. 2022) and other taxa (e.g., Arnaud et al. 2012). One potential caveat is that the home ranges of Grasshopper Sparrows can be substantially larger than their territory area (Hannah et al. 2024), so if they are spending substantial amounts of time foraging outside of their home pasture, it is possible that pasture‐specific metrics of food availability would not reflect their foraging landscape. The metabolite profiles would reflect foraging dynamics, irrespective of the location of that foraging, so ultimately, our conclusions are not influenced by the difference between territory and home range in this system.

Unlike the systems in which food has been implicated in dispersal movements, temperate grasslands may be somewhat atypical. These are highly productive and highly seasonal environments that, for a short period each growing season, may support a far greater number of arthropod prey than consumers could routinely exhaust. Indeed, the idea that arthropod prey is “superabundant” in grassland ecosystems is not a new one (Wiens 1974). Additionally, populations of grassland birds have continued to decline dramatically over the past 50 years (Rosenberg et al. 2019), which would further reduce competition for food unless prey has declined more rapidly than their predators (Mahony et al. 2022; Bernath‐Plaisted et al. 2023). Certainly, declines of insect prey (due to multiple anthropogenic stressors) are a threat to insectivorous species around the world (Wagner et al. 2021). However, declines in standardized, long‐term ecological data from the United States including at the Konza (Crossley et al. 2020) and elsewhere in Great Plains grasslands (Skipper et al. 2020) fail to demonstrate the alarming declines evident elsewhere. Thus, the lack of evidence for food driving the dispersal of the birds in our study may well be a general reflection of distinct processes influencing the ecology of grassland systems compared to forested environments.

This study provides a sobering example of the importance of challenging common assumptions about the drivers of animal behavior. In the animal movement and habitat quality literature, there is a pervasive assumption that relationships documented by empirical studies reflect underlying variation in food availability (e.g., Rushing et al. 2015; Norevik et al. 2019). While it is frequently possible such assumptions are true, they are rarely tested against alternative explanations that could either reveal associations between animals and alternate factors shaping their behavior, or more complex, multi‐causal explanations. Failure to get those associations right means that any population forecasting and management actions will likely be incorrect or ineffective. Additionally, inferences to community‐level processes will be based on flawed assumptions. There have been many, repeated calls for the application of strong inference in Ecology and other fields (Betini et al. 2017; Sells et al. 2018); the importance of this approach has never been greater in the current conservation crisis.

## Author Contributions

**Emily J. Williams:** conceptualization (equal), formal analysis (equal), investigation (lead), writing – original draft (equal), writing – review and editing (supporting). **Theo K. Michaels:** formal analysis (equal), visualization (lead), writing – review and editing (supporting). **W. Alice Boyle:** conceptualization (lead), data curation (lead), funding acquisition (lead), investigation (equal), methodology (equal), project administration (lead), supervision (lead), writing – original draft (equal), writing – review and editing (lead).

## Funding

This work was supported by Friends of the Sunset Zoo, Division of Environmental Biology (DEB‐1440484, DEB‐1754491), Wilson Ornithological Society, Kansas State University, University of Western Ontario, and The Nature Conservancy.

## Ethics Statement

All research was conducted under approved ethical animal care and use protocols (Kansas State University no. 3260) and permits from the North American Bird Banding Laboratory (#23836), Konza Prairie Biological Station (#202 and 254), and the Kansas Department of Wildlife, Parks, and Tourism (SC‐106‐2013, SC‐110‐2014, and SC‐037‐2015).

## Conflicts of Interest

The authors declare no conflicts of interest.

## Supporting information


**Table S1:** Summary of modeling approach, response and explanatory variables, and random effects in analyses of each of the five predictions tested in this paper.
**Table S2:** Prediction *i* model results: results of linear mixed model explaining variation in edible mass of arthropod prey available to Grasshopper Sparrows.

## Data Availability

All data and code to reproduce results in this manuscript are archived on the Zenodo repository (https://doi.org/10.5281/zenodo.21707477). Complete data on captures, locations, and individual metrics of the birds used in this and related studies are available through the Konza Prairie LTER Data Portal (Boyle 2023) and are archived as: https://portal.edirepository.org/nis/mapbrowse?packageid=knb‐lter‐knz.111.4.
