# Supplementary material for: Food Availability Cannot Explain Dispersal and Settlement Decisions of a Grassland Bird
Source: Ecol Evol. 2026 Aug 9;16(8):e74173. doi: 10.1002/ece3.74173 (PMC13454223; doi:10.1002/ece3.74173)
Supplement: Supplementary file 1 [file ECE3-16-e74173-s001.pdf]

Supplemental Materials for

Food availability cannot explain dispersal and settlement decisions of a grassland bird

Emily J. Williams[1,2], Theo K. Michaels[1], and W. Alice Boyle[1,3]

1. Division of Biology, Kansas State University, Manhattan, KS, USA

2. Current address: Georgetown University, Washington, DC, USA

3. Current address: Biology Department, University of Western Ontario, London, ON, Canada

corresponding author: aboyle7@uwo.ca

This PDF file includes:

Supplemental Table S1, Supplemental Table S2

Table S1 Summary of modeling approach, response and explanatory variables, and random effects in analyses of each of the five predictions tested in this paper.

Prediction	Model Type	Response ~ Explanatory Variables	Radom Factors
i	LMM	Edible Biomass ~ Time Period + Grazing Treatment + Year Since Burned	1|Pasture + 1|Pasture:TransectID
i	LMM interaction	Edible Biomass ~ Time Period + Grazing Treatment + Year Since Burned + Time Period*Grazing Treatment + Time Period*Year Since Burned	1|Pasture + 1|Pasture:TransectID
ii	LM	Conc. TRIG ~ Capture Time + Capture Date + Fat Score + Sex	NA
ii	LM w/Edible Biomass	Conc. TRIG ~ Capture Time + Capture Date + Fat Score + Sex + Edible Biomass	NA
iii	LM	Density ~ Resid TRIG	NA
iii	LM w/Time Period	Density ~ Resid TRIG + Time Period	NA
iii	LM interaction	Density ~ Resid TRIG + Time Period + Resid TRIG*Time Period	NA
iv	LM	Density ~ Edible Biomass	NA
iv	LM w/Time Period	Density ~ Edible Biomass + Time Period	NA
iv	LM interaction	Density ~ Edible Biomass + Time Period + Edible Biomass*Time Period	NA
v	t test	Diff. in ResidTRIG ~ Dispersal Status	NA

Table S2 Prediction *i* model results: results of linear mixed model explaining variation in edible mass of arthropod prey available to Grasshopper Sparrows

	**Model 1: additive & interactive**	**Model 2: additive**
AICc*	311.09	330.14
ΔAICc[§]	0	19.05
AICcWt[†]	1	0

Model 1 fit: AIC = 306.3, log likelihood = -135.2.0, deviance = 270.3; residual df = 144, conditional R^2 = 0.74, marginal R^2 = 0.57

**Parameter**	*Estimate (Std. Error)*	
Mid period (relative to early)	2.01 (0.21)	1.10 (0.11)
Late period (relative to early)	2.24 (0.21)	1.48 (0.11)
Cattle grazing (relative to bison)	0.54 (0.31)	0.03 (0.27)
No grazing (relative to bison)	0.33 (0.31)	-0.12 (0.27)
1 year-since-burn (relative to 0)	0.12 (0.28)	-0.37 (0.24)
2 year-since-burn (relative to 0)	-0.07 (0.40)	-0.69 (0.35)
Mid period * Cattle	-0.95 (0.26)	
Late period * Cattle	-0.59 (0.26)	
Mid period * No grazing	-0.81 (0.26)	
Late period * No grazing	-0.54 (0.26)	
Mid period * 1 yr since burn	-0.56 (0.24)	
Late period * 1 yr since burn	-0.93 (0.24)	
Mid period * 2 yr since burn	-0.84 (0.34)	
Late period * 2 yr since burn	-1.02 (0.34)	
**Random effect**	*Variance (Std. Deviation)*	

Pasture	0.14 (0.37)	0.13 (0.36)
Pasture:Transect ID	0.02 (0.13)	0.00 (0.00)

* Akaike's Information Criterion corrected for small sample size

§ Difference in AIC_C between top model (1) and other models. Differences >4 generally interpreted as strong evidence for distinguishing fit of models to data

† The relative likelihood that the model is the best among a set of candidate models, with a weight of 1.0 being the most likely
